# Supplementary material for: ProteinShader: illustrative rendering of macromolecules
Source: BMC Struct Biol. 2009 Mar 30;9:19. doi: 10.1186/1472-6807-9-19 (PMC2672931; doi:10.1186/1472-6807-9-19)
Supplement: Additional file 1 — ProteinShader program without source code. This compressed file contains the complete ProteinShader program including associated libraries, but no source code. A README.txt file gives an overview of the ProteinShader distribution, and the index.html file in the help subdirectory has directions on getting started with the program as well as a set of tutorials. [file 1472-6807-9-19-S1.zip › ProteinShader-beta-0_9_4-binary/help/api/org/proteinshader/structure/enums/class-use/HelixEnum.html]

Uses of Class org.proteinshader.structure.enums.HelixEnum (ProteinShader API)


|  |  |  |
| --- | --- | --- |
| **Overview** \| **Package** \| **Class** \| **Use** \| **Tree** \| **Deprecated** \| **Index** \| **Help** | |  |
| PREV   NEXT | **FRAMES**    **NO FRAMES**     **All Classes** |  |


---


## **Uses of Class org.proteinshader.structure.enums.HelixEnum**

| Packages that use HelixEnum | |
| --- | --- |
| **org.proteinshader.structure** | Holds the classes that store information from a Protein Data Bank file: Structure, Model, Chain, AminoAcid, Heterogen, Water, Atom, Bond, Helix, BetaStrand, Loop, *etc*. |
| **org.proteinshader.structure.enums** | Holds the enums needed by org.proteinshader.structure and its subpackages. |
| **org.proteinshader.structure.io** | Holds the classes needed for reading Protein Data Bank structure files. |

Uses of HelixEnum in org.proteinshader.structure

| Fields in org.proteinshader.structure declared as HelixEnum | |
| --- | --- |
| `static HelixEnum` | `Helix.DEFAULT_TYPE`             The default Helix type is RH\_ALPHA. |

| Methods in org.proteinshader.structure that return HelixEnum | |
| --- | --- |
| `HelixEnum` | `Helix.getType()`             Returns the type of Helix. |

| Methods in org.proteinshader.structure with parameters of type HelixEnum | |
| --- | --- |
| `Helix` | `Chain.addNewHelix(String helixID, String serialNo, String startResidueID, String endResidueID, HelixEnum type)`             Creates a new Helix with the Helix ID given as an argument, adds the Helix to the Chain's collection of Helices, and returns a reference to the new Helix. |
| `void` | `Structure.addNewHelix(String chainID, String helixID, String serialNo, String startResidueID, String endResidueID, HelixEnum type)`             The Helix will be added to the equivalent Chain on each Model, but this addition will only work if the sequence of Residues that the Helix refers to have already been added. |
| `void` | `Model.addNewHelix(String chainID, String helixID, String serialNo, String startResidueID, String endResidueID, HelixEnum type)`             The Helix will be added to the Chain only if the sequence of Residues that the Helix refers to has already been added. |
| `void` | `Helix.setType(HelixEnum type)`             Sets the type of Helix. |

Constructors in org.proteinshader.structure with parameters of type HelixEnum

`Helix(String helixID,
String serialNo,
String startResidueID,
String endResidueID,
Chain chain,
HelixEnum type,
HelixShapeEnum shape)`
  
          Constructs a Helix.

Uses of HelixEnum in org.proteinshader.structure.enums

| Methods in org.proteinshader.structure.enums that return HelixEnum | |
| --- | --- |
| `static HelixEnum` | `HelixEnum.valueOf(int pdbClassNo)`             Returns the HelixEnum corresponding to the PDB classification number given as an argument. |
| `static HelixEnum` | `HelixEnum.valueOf(String name)`             Returns the enum constant of this type with the specified name. |
| `static HelixEnum[]` | `HelixEnum.values()`             Returns an array containing the constants of this enum type, in the order they're declared. |

Uses of HelixEnum in org.proteinshader.structure.io

| Methods in org.proteinshader.structure.io that return HelixEnum | |
| --- | --- |
| `HelixEnum` | `PDBHelixFieldExtractor.extractType(String line)`             Extracts the helix type from (helixClass field: 39-40). |
| `HelixEnum` | `HelixRecord.getType()`             Returns the type of Helix. |

Constructors in org.proteinshader.structure.io with parameters of type HelixEnum

`HelixRecord(String chainID,
String helixID,
String serialNo,
String startResidueID,
String endResidueID,
HelixEnum type)`
  
          Constructs a HelixRecord.

---


|  |  |  |
| --- | --- | --- |
| **Overview** \| **Package** \| **Class** \| **Use** \| **Tree** \| **Deprecated** \| **Index** \| **Help** | |  |
| PREV   NEXT | **FRAMES**    **NO FRAMES**     **All Classes** |  |


---

# *Copyright © 2007-2008*
